# Developing an Injectable Nanofibrous Extracellular Matrix Hydrogel With an Integrin αvβ3 Ligand to Improve Endothelial Cell Survival, Engraftment and Vascularization

**DOI:** 10.3389/fbioe.2020.00890

**Published:** 2020-07-29

**Authors:** Dake Hao, Ruiwu Liu, Kewa Gao, Chuanchao He, Siqi He, Cunyi Zhao, Gang Sun, Diana L. Farmer, Alyssa Panitch, Kit S. Lam, Aijun Wang

**Affiliations:** ^1^Department of Surgery, School of Medicine, University of California, Davis, Sacramento, CA, United States; ^2^Institute for Pediatric Regenerative Medicine, Shriners Hospitals for Children, Sacramento, CA, United States; ^3^Department of Biochemistry and Molecular Medicine, School of Medicine, University of California, Davis, Sacramento, CA, United States; ^4^Department of Biological and Agricultural Engineering, University of California, Davis, Davis, CA, United States; ^5^Department of Biomedical Engineering, University of California, Davis, Davis, CA, United States

**Keywords:** collagen hydrogel, endothelial cell, integrin-based ligand, cell engraftment, tissue regeneration

## Abstract

Endothelial cell (EC) transplantation via injectable collagen hydrogel has received much attention as a potential treatment for various vascular diseases. However, the therapeutic effect of transplanted ECs is limited by their poor viability, which partially occurs as a result of cellular apoptosis triggered by the insufficient cell-extracellular matrix (ECM) engagement. Integrin binding to the ECM is crucial for cell anchorage to the surrounding matrix, cell spreading and migration, and further activation of intracellular signaling pathways. Although collagen contains several different types of integrin binding sites, it still lacks sufficient specific binding sites for ECs. Previously, using one-bead one-compound (OBOC) combinatorial technology, we identified LXW7, an integrin αvβ3 ligand, which possessed a strong binding affinity to and enhanced functionality of ECs. In this study, to improve the EC-matrix interaction, we developed an approach to molecularly conjugate LXW7 to the collagen backbone, via a collagen binding peptide SILY, in order to increase EC specific integrin binding sites on the collagen hydrogel. Results showed that in the *in vitro* 2-dimensional (2D) culture model, the LXW7-treated collagen surface significantly improved EC attachment and survival and decreased caspase 3 activity in an ischemic-mimicking environment. In the *in vitro* 3-dimensional (3D) culture model, LXW7-modified collagen hydrogel significantly improved EC spreading, proliferation, and survival. In a mouse subcutaneous implantation model, LXW7-modified collagen hydrogel improved the engraftment of transplanted ECs and supported ECs to form vascular network structures. Therefore, LXW7-functionalized collagen hydrogel has shown promising potential to improve vascularization in tissue regeneration and may be used as a novel tool for EC delivery and the treatment of vascular diseases.

## Introduction

Endothelial cell (EC) transplantation has been widely used for the treatment of various types of vascular diseases, such as myocardial ischemia, cerebrovascular disease, and peripheral vascular disease (Fadini et al., 2005; Singhal et al., 2010; Blum et al., 2012). However, exploration and tracking of EC behavior and fate after transplantation has indicated that ECs exhibit poor survival and engraftment rates, which proves to be the crucial limitation of EC transplantation (Hall and Jevnikar, 2003). To overcome this limitation, different approaches, such as cell pre-conditioning, genetic modification and co-transplantation (Penn and Mangi, 2008; Yu et al., 2013; Sun et al., 2016; Shafiee et al., 2017; Hao et al., 2019), have been used to improve EC engraftment after transplantation. However, these approaches have raised safety concerns and have complicated regulatory pathways. Thus, there is still an urgent need to develop safe and easy-for-translation approaches for EC transplantation that allow for improved cell engraftment rates.

Biomaterial carriers are attractive tools for improving cell delivery and survival (Qi et al., 2015). Hydrogel systems are currently widely studied in the field of stem cell transplantation, due to their 3-dimensional (3D) cross-linked networks, cytocompatibility, injectability, and biocompatibility (Mulyasasmita et al., 2014; Robinson et al., 2016; Hao et al., 2020b). Collagen is the main structural protein in natural extracellular matrix (ECM), which can mimic the physical characteristics of ECM (Di Lullo et al., 2002). Particularly, type I collagen has great potential as a cell delivery medium, because it is ubiquitous and can self-assemble under physiological conditions (Glowacki and Mizuno, 2008; Copes et al., 2019). Integrins are heterodimeric transmembrane receptors present on the cell surface (Giancotti and Ruoslahti, 1999). Upon ligand binding, integrins facilitate cell-cell and cell-ECM adhesion, activate signal transduction pathways, and regulate cell functions (Caiado and Dias, 2012; Malinin et al., 2012). Integrins have been shown to play critical roles in cartilage (Liang et al., 2015), bone (Kundu et al., 2009), neuronal (Chen et al., 2018), pancreatic (Krishnamurthy and Wang, 2009) and vascular (Li et al., 2017) regeneration. Consequently, increasing specific integrin binding sites will be significant in advancing the development of an engineered hydrogel cell delivery system for use in tissue regeneration applications. Although collagen has several integrin binding sites (Xu et al., 2000; Znoyko et al., 2006; Zeltz and Gullberg, 2016), such as α1β1, α2β1, α10β1, and α11β1, it lacks sufficient EC specific integrin binding sites. According to previous studies, αvβ3 integrin expressed on ECs has been shown to be crucial for EC adhesion and for advancing angiogenic development (Tzima et al., 2001; Sheppard, 2002; Avraamides et al., 2008). Therefore, identifying an integrin αvβ3 ligand specifically bound to ECs and using it to engineer the collagen hydrogel will be valuable for EC transplantation in tissue regeneration applications.

One-bead one-compound (OBOC) combinatorial technology is an ultra-high throughput chemical library synthesis and screening method, which is suitable for integrin-based ligand discovery (Lam et al., 1991). Previously, we have identified various potent ligands, such as LXY30, LXW7, and LLP2A targeting integrins α3β1, αvβ3, and α4β1, respectively, by employing the OBOC combinatorial technology (Peng et al., 2006; Yao et al., 2009; Xiao et al., 2010). We have also found that LXW7 had specific binding to ECs via αvβ3 integrin and biomaterial scaffolds decorated with LXW7 was found to improve EC adhesion and functions *in vitro* and promoted vascularization in a rat carotid artery bypass vascular graft model (Hao et al., 2017, 2020a). Thus, LXW7 will be an ideal choice to modify collagen hydrogel for increasing the number of EC specific integrin binding sites. SILY peptide, RRANAALKAGELYKSILY, is a high-affinity collagen binding ligand derived from platelet membrane receptors that bind to α1 chains in collagen (Paderi and Panitch, 2008; Goldbloom-Helzner et al., 2019). SILY has also been conjugated to other functional molecules and used in medical applications related to collagen (Paderi et al., 2011; Scott et al., 2013). Therefore, in this study, we propose to molecularly immobilize LXW7 onto collagen backbone by using SILY as the junction to increase the EC specific integrin binding sites of collagen hydrogel to improve EC binding, survival and ultimately engraftment after transplantation.

## Materials and Methods

### Cell Culture

We used endothelial colony forming cell (ECFC) banks as described in our previous study (Hao et al., 2017, 2020a). ECFCs were expanded and cultured in Endothelial Cell Growth Medium-2 BulletKit medium (EGM-2, Lonza). ECFCs between P3 and P6 were used for all experiments.

### ECFC Attachment on LXW7-Treated Collagen Surface

**FIGURE 1:**
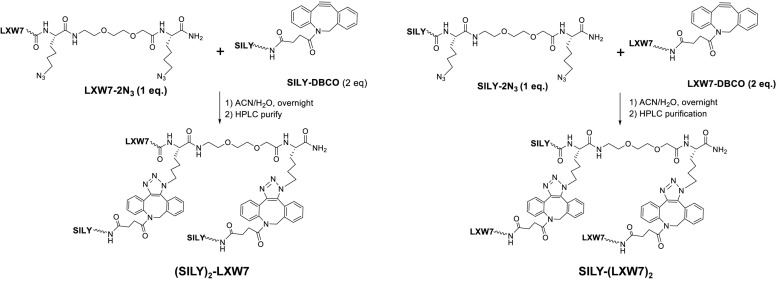
Chemical synthesis of (SILY)_2_-LXW7 or SILY-(LXW7)_2._

To facilitate the LXW7 modification on 2-dimensional (2D) collagen culture surface and 3-dimensional (3D) collagen hydrogel, we synthesized (SILY)_2_-LXW7 and SILY-(LXW7)_2_ (Figure 1) through three steps: 1) standard solid phase peptide synthesis (SPPS) of LXW7-2N_3_ or SILY-2N_3_, 2) SPPS synthesis of SILY-DBCO or LXW7-DBCO, 3) DBCO-azido copper-free Click conjugation by mixing LXW7-2N_3_ with 2 eq. of SILY-DBCO or SILY-2N_3_ with 2 eq. of LXW7-DBCO, respectively. Detailed synthesis was described in Supplementary Figure S1. To modify the collagen culture surface with LXW7, target culture wells in a 24-well plate were coated with 500 μL of 100 ng/mL collagen type I (PureCol) and incubated for 1 h at 37 °C. Collagen coated wells were rinsed three times with PBS (HyClone) and were treated with 500 μL 20 μM of (SILY)_2_-LXW7 or SILY-(LXW7)_2_. After 1 h, the wells were washed three times with PBS and blocked with 1% BSA (Thermo Fisher Scientific) for 1 h. After, the wells were rinsed three times with PBS. For the cell attachment assay, 5 × 10^3^ ECFCs were added to the wells and incubated for 5 min at 37°C and 5% CO_2_. The wells were washed three times with PBS, and the adhered cells were fixed in 10% formalin (Azer Scientific) for 20 min. The wells were washed, and then nuclei were stained with 4′, 6-diamidino-2-phenylindole (DAPI, Sigma). After three washings with PBS, the ECFCs were imaged using a Carl Zeiss Axio Observer D1 inverted microscope. Image quantification was performed using the ImageJ software (NIH).

### ECFC Apoptosis and Survival on LXW7-Treated Collagen Surface Under Ischemic-Mimicking Hypoxic Environment

The ischemic-mimicking hypoxic environment was set up as cells cultured in EGM-2 with 1% fetal bovine serum (FBS) and low concentration growth factors (1/10 of the original bulk in the EGM-2 bullet kit) at 1% O_2_, 37°C and 5% CO_2_. ECFCs were seeded in 96-well plates treated with collagen, collagen and (SILY)_2_-LXW7, or collagen and SILY-(LXW7)_2_. For caspase 3 assay, the cells were cultured in ischemic-mimicking hypoxic environment for 6 h, then lysed and analyzed by using a Caspase 3 Assay Kit (Cell Signaling Technology) according to the manufacturer’s instruction. Fluorescence (ex 380 nm/em 450 nm) was measured using a SpectraMax i3x Multi-Mode Detection Platform (Molecular Devices). For the cell survival assay, the ECFCs were cultured for 5 d and determined using a CellTiter 96^®^ AQueous One Solution Cell Proliferation Assay (MTS, Promega) according to the manufacturer’s instruction. The amount of soluble formazan product produced by the reduction of MTS by metabolically active cells was measured at the 490 nm absorbance using the SpectraMax i3x Multi-Mode Detection Platform.

### Preparation and Characterization of LXW7-Modified Collagen Hydrogel

For optimization of collagen hydrogel concentration, the original 10 mg/mL collage type I was diluted to 1, 2, 4, and 8 mg/mL with PBS, respectively. The same number of ECFCs was loaded into the collagen hydrogel with different concentration and cultured at 37°C, 5% CO_2_ for 5 days, then the number of cells was determined using MTS as described above. For the preparation and evaluation of LXW7-modified collagen hydrogel, the diluted 2 mg/mL collagen was mixed with SILY-(LXW7)_2_ at the different concentration of SILY-(LXW7)_2_/collagen (nmol/mg), such as 0, 0.005, 0.05, 0.25, 0.5, 2.5, 5, 12.5, or 25 nmol/mg. The LXW7-modified collagen (0.1 mL) was then put in 48-well plates and incubated at 37°C for 1 h, then 100 μL PBS was added into the wells. After 24 h, the PBS was collected for High-performance liquid chromatography (HPLC) analysis to quantify the amount of unbound free SILY-(LXW7)_2_ that was eluted to the solution. Briefly, HPLC analysis was performed on the Waters 2996 HPLC system equipped with a Waters XTerra^®^ MS column (5 μm, C18, 150 × 4.6 mm). A linear gradient was run from 100% solution A (water/0.1% trifluoroacetic acid) to 100% solution B (acetonitrile/0.1% trifluoroacetic acid) within 20 min with a flow rate at 1.0 mL/min. The UV detection wavelength was 214 nm. The compressive modulus of the untreated collagen hydrogel and the LXW7-modified collagen hydrogel were determined using the Instron 5566 Universal Testing Machine.

### Lentiviral Vector Transduction

All lentiviral constructs were generated at the UC Davis Institute for Regenerative Cures (IRC) Vector Core. To track the cell fate and behavior *in vitro* and *in vivo*, ECFCs were transduced with the pCCLc-MNDU3-LUC-PGK-EGFP-WPRE vector as previously described (Kumar et al., 2018; Gao et al., 2019, 2020; Rose et al., 2020). Transduction was performed in transduction medium consisting of DMEM high glucose (HyClone), 10% FBS (HyClone), and 8 μg/mL protamine sulfate (MP Biomedicals) for 6 h. The vectors were transduced at a multiplicity of infection (MOI) of 10. After that, ECFCs were cultured in EGM-2 medium for 72 h. After 72 h, ECFCs were screened for neomycin resistance for 7 days cultured in medium containing 2 μg/mL of G418 (EMD, Millipore). Successfully transduced ECFCs were then cultured and expanded in EGM-2 medium.

### ECFC Behavior in LXW7-Modified Collagen Hydrogel

For cell sprouting assay, ECFCs were loaded in the untreated collagen hydrogel, or LXW7-modified collagen hydrogel, respectively, and cultured in EGM-2 for 3 d. Images were taken at day 1 and day 3 by using the Carl Zeiss Axio Observer D1 inverted microscope. Image quantification was performed using the ImageJ software. For capillary network formation, three groups were set up: (1) ECFCs cultured in untreated collagen hydrogel, (2) ECFCs cultured in LXW7-modified collagen hydrogel, (3) ECFCs incubated with a monoclonal anti-αvβ3 integrin blocking antibody (MAB1876, Millipore) first to block the integrin αvβ3 expressed on the ECFCs, then cultured in the LXW7-modified collagen hydrogel. Images were taken at day 5 by using the Carl Zeiss Axio Observer D1 inverted microscope. Image quantification was performed using the ImageJ software. For cell proliferation, ECFCs were loaded in the untreated collagen hydrogel or LXW7-modified collagen hydrogel, respectively, and cultured in EGM-2 at 37°C, 20% O_2_ and 5% CO_2_ for 5 days. Cell metabolic activity and proliferation was quantified every 24 h using MTS as described above. For cell survival, ECFCs were loaded in the untreated collagen hydrogel or LXW7-modified collagen hydrogel, respectively, and cultured under ischemic-mimicking hypoxic environment for 5 days. Cell metabolic activity was quantified every 24 h using MTS, as described above.

### *In vivo* Cell Transplantation

All animal procedures were approved by the Institutional Animal Care and Use Committee at the University of California, Davis. All facilities used during the study period were accredited by the Association for the Assessment and Accreditation of Laboratory Animal Care International (AAALAC). NSG (NOD/SCID/ IL2Rγ^–/–^) immunodeficient mice were purchased from The Jackson Laboratory. 5 × 10^5^ transduced ECFCs were loaded in 500 μL untreated collagen hydrogel or LXW7-modified collagen hydrogel, respectively, and injected subcutaneously to the groin at both sides of each mouse.

### Bioluminescence Imaging

Cells transplanted in NSG mice were monitored via the *In Vivo* Imaging Spectrum (IVIS) system (PerkinElmer) as previously described (Kumar et al., 2018; Gao et al., 2019; Rose et al., 2020). Briefly, animals were injected intraperitoneally with luciferase substrate D-luciferin (Gold Biotechnology) at 100 mg/kg body weight and maintained under anesthesia with 2% inhaled isoflurane for 5 min before imaging. The transplanted NSG mice were imaged at the day of transplantation (week 0) and weekly thereafter for up to 10 weeks after transplantation. Images were analyzed by using Living Image^®^2.50 (Perkin Elmer). Total intensity was measured within a defined area of the signal. Baseline intensity was determined by using the same defined area where there is no positive signal in the same animal.

### Immunohistochemistry

Samples were collected, fixed with 4% paraformaldehyde for 24 h, protected by 30% sucrose dehydration for 48 h, and embedded in the O.C.T compound (Sakura Finetek USA, Inc). Serial sections were made at the thickness of 15 μm using a Cryostat (Leica CM3050S) and collected onto microscope slides (Matsunami Glass). Tissue sections were extensively washed with PBS, blocked with 5% BSA in PBS at room temperature for 1 h, and stained with primary antibody at 4°C overnight. The dilution of primary antibody was goat anti-GFP (Novus Biologicals) at 1:100. Sections were incubated with secondary antibodies diluted at 1:500 for 1 h at room temperature. The secondary antibody was donkey anti-goat (Thermo Fisher Scientific) conjugated with Alexa488. The slides were counterstained with 1:5000 dilution of DAPI for 5 min, mounted with Prolong Diamond Antifade Mountant (Invitrogen), and imaged with the Zeiss Observer Z1 microscope.

### Statistical Analysis

For two-sample comparison, a student’s *t*-test was used. For multiple-sample comparison, analysis of variance (ANOVA) was performed to detect whether a significant difference existed between groups with different treatments. A *p*-value of 0.05 or less indicates a significant difference between samples in comparison.

## Results and Discussion

### LXW7-Treated Collagen Surface Improved ECFC Attachment

**FIGURE 2:**
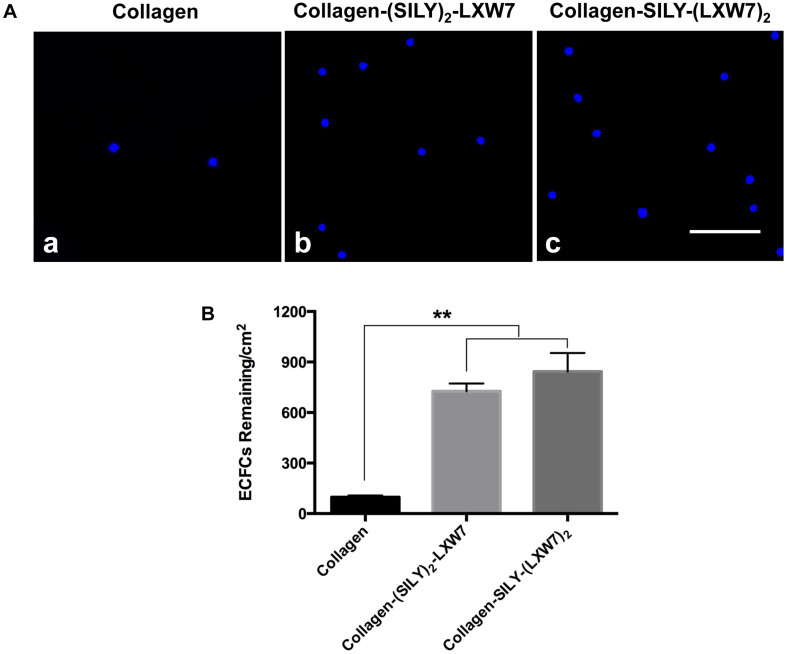
Attachment of ECFCs on LXW7-treated collagen surface. **(A)** Images of ECFC attachment on untreated collagen surface **(a)**, (SILY)_2_-LXW7 treated collagen surface **(b)** and SILY-(LXW7)_2_ treated collagen surface **(c)**. Scale bar = 100 μm. **(B)** Quantification of the number of ECFCs attached on the different treated surfaces. Data were expressed as mean ± standard deviation: ^∗∗^*p* < 0.01 (*n* = 5).

Natural ECM displays numerous copies of several cell binding sites to support cell-matrix adhesion that is crucial for cell functions (Werb, 1997; Yue, 2014). Cell-matrix adhesion is formed through the utilization of cell adhesion molecules that bind to the cell surface (Zhong and Rescorla, 2012). Integrins are a very important class of cell adhesion heterodimer molecules, comprised of two subunits that facilitate attachments between the cell surface and the ECM, and can greatly influence cell behavior (Gumbiner, 1996). Although collagen is the main component of natural ECM, it still lacks specific EC adhesion sites. Our previous study demonstrated an integrin αvβ3 ligand LXW7 possessed specific binding to ECs (Hao et al., 2017). Thus, to increase ECFC binding sites that facilitate ECFC attachment on the collagen surface and evaluate density effects of binding sites on ECFC functions, we synthesized two different types of SILY-LXW7 derived compounds, (SILY)_2_-LXW7 and SILY-(LXW7)_2_, that can be used conveniently to conjugate LXW7 onto the collagen backbone. The results showed that cell culture surface treated with both (SILY)_2_-LXW7 and SILY-(LXW7)_2_ significantly improved ECFC attachment compared to the untreated collagen surface (Figure 2). The SILY-(LXW7)_2_ treated collagen surface supported more ECFC attachment, compared to the (SILY)_2_-LXW7 treated collagen surface indicating that a higher density of LXW7 was more beneficial for ECFC attachment (Figure 2).

### LXW7-Treated Collagen Surface Suppressed ECFC Apoptosis and Improved ECFC Survival Under Ischemic-Mimicking Hypoxic Environment

**FIGURE 3:**
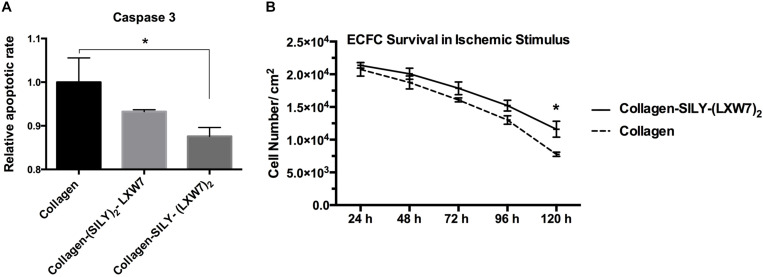
Apoptosis and survival of ECFCs on LXW7-treated collagen surface. **(A)** Quantification of caspase 3 expression of ECFCs cultured on different treated surfaces under ischemic-mimicking hypoxic environment. **(B)** Quantification of survival of ECFCs cultured on the different treated surfaces under ischemic-mimicking hypoxic environment. Data were expressed as mean ± standard deviation: ^∗^*p* < 0.05 (*n* = 5).

Cell-matrix interaction regulates cellular homeostasis in multiple ways (Almeida et al., 2000; Niit et al., 2015). Disruption of this connection has deleterious effects on cell binding and survival, which leads to a specific type of apoptosis known as anoikis in most cell types (Reddig and Juliano, 2005). Caspase-3 is responsible for chromatin condensation and DNA fragmentation that is necessary in apoptosis (Porter and Janicke, 1999). The results showed both (SILY)_2_-LXW7 and SILY-(LXW7)_2_ treated collagen surfaces suppressed expression of caspase 3 in ECFCs, compared to the collagen surface under the ischemic-mimicking hypoxic environment, and only SILY-(LXW7)_2_ treated collagen surface significantly decreased the expression of caspase 3 in ECFCs, compared to the collagen surface (Figure 3A). These results may be caused by the different binding sites provided by (SILY)_2_-LXW7 or SILY-(LXW7)_2._ Stoichiometrically, the LXW7 binding sites provided by SILY-(LXW7)_2_ was four times greater compared to the LXW7 binding sites provided by (SILY)_2_-LXW7. Subsequently, the results showed that the SILY-(LXW7)_2_ treated collagen surface significantly improved ECFC survival under the ischemic-mimicking hypoxic environment (Figure 3B), indicating that the LXW7-treated collagen surface was beneficial for ECFC survival by increasing the number of ECFC specific integrin binding sites.

### Evaluation of LXW7 Conjugation on Collagen Hydrogel

**FIGURE 4:**
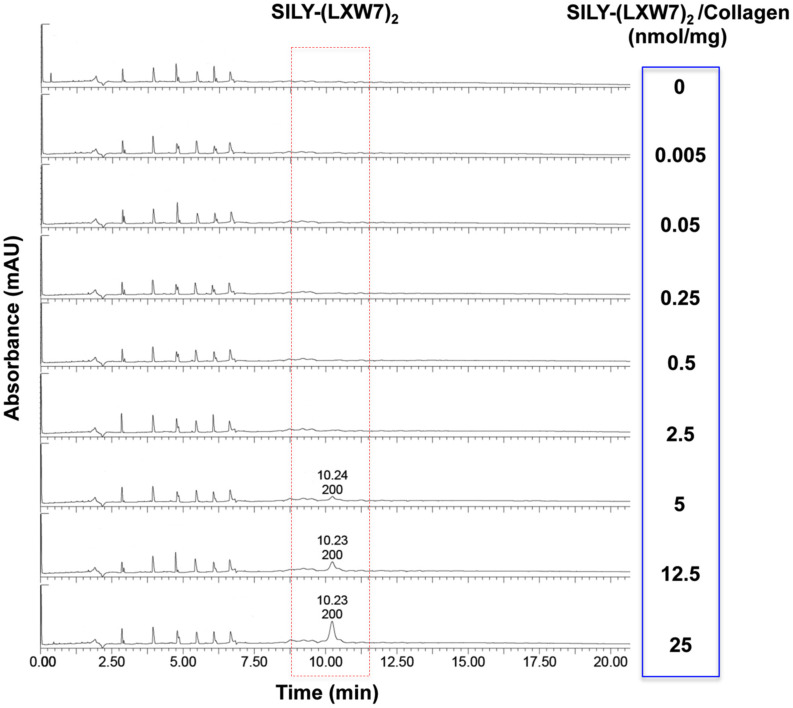
HPLC evaluation of LXW7 conjugation on collagen hydrogel. The elution of unbound free SILY-(LXW7)_2_ into the incubating PBS was quantified using HPLC. The peak of SILY-(LXW7)_2_ was not shown until the amount of SILY-(LXW7)_2_ used to modify collagen hydrogel was up to 5 nmol/mg, and the peaks increased as the amount of SILY-(LXW7)_2_ increased.

The effect of ECM stiffness on cell biology, signaling and response has been well-established, which has significant implications for tissue regeneration (Antoine et al., 2014). Particularly, ECM stiffness has also been linked with endothelial integrity and consequently regulation of angiogenesis (Sieminski et al., 2004). The concentrations of hydrogel could significantly influence cell behavior and growth, due to their physical properties such as stiffness (Banerjee et al., 2009; Ahearne, 2014). Before LXW7 modification, we identified the optimal concentration of collagen hydrogel for ECFC growth. The results showed that both 1 mg/mL and 2 mg/mL collagen hydrogel significantly improved ECFC growth, compared to 4 mg/mL and 8 mg/mL collagen hydrogel. There was no significant difference between the 1 mg/mL and 2 mg/mL collagen hydrogel (Supplementary Figure S2). In addition, to consider the difference in solidification time, we chose 2 mg/mL as the optimal concentration of collagen hydrogel for the following tests. For the LXW7 modification on collagen hydrogel, different concentration of SILY-(LXW7)_2_ were used to modify the collagen hydrogel. HPLC has been widely used to separate and analyze the sample mixture in a discrete small volume (Gerber et al., 2004). The HPLC results did not show any peak of SILY-(LXW7)_2_ until the amount of SILY-(LXW7)_2_ used to modify collagen hydrogel was up to 5 nmol/mg, and the peaks increased as the amount of SILY-(LXW7)_2_ increased (Figure 4). These results demonstrated that LXW7 had been successfully conjugated onto the collagen hydrogel via the “SILY-collagen” binding approach, and the saturation concentration of modification was between 2.5 and 5 nmol/mg. Compressive modulus of the collagen hydrogel before and after LXW7 modification showed no significant difference (Supplementary Figure S3) indicating the SILY-(LXW7)_2_ modification approach did not significantly change the stiffness of the collagen hydrogel. This was probably because only one end of the SILY-(LXW7)_2_ molecule carried the –SILY group that could bind to collagen, and the –(LXW7)_2_ end could not bind to collagen, therefore the SILY-(LXW7)_2_ modification approach did not induce molecular cross-linking of collagen.

### LXW7-Modified Collagen Hydrogel Improved ECFC Sprouting and Promoted ECFC Vascular Network Formation by Increasing Integrin αvβ3 Binding Sites

**FIGURE 5:**
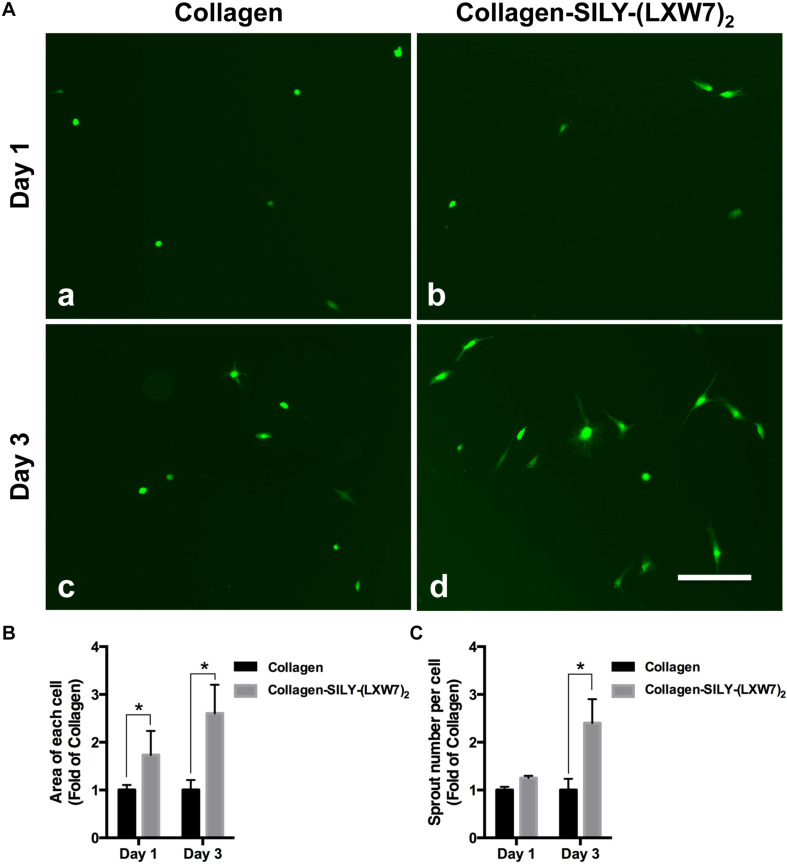
ECFC spreading and spouting in LXW7-modified collagen hydrogel. **(A)** Images of ECFC morphology in untreated collagen hydrogel **(a)** and LXW7-modified collagen hydrogel **(b)** after 1 day culture and ECFC morphology in untreated collagen hydrogel **(c)** and LXW7-modified collagen hydrogel **(d)** after 3 day culture. Scale bar = 100 nm. **(B)** Quantification of the cell area of ECFCs cultured in collagen hydrogel with different modification. **(C)** Quantification of the number of sprouts of ECFCs cultured in collagen hydrogel with different modification. Data were expressed as mean ± standard deviation: ^∗^*p* < 0.05 (*n* = 5).

**FIGURE 6:**
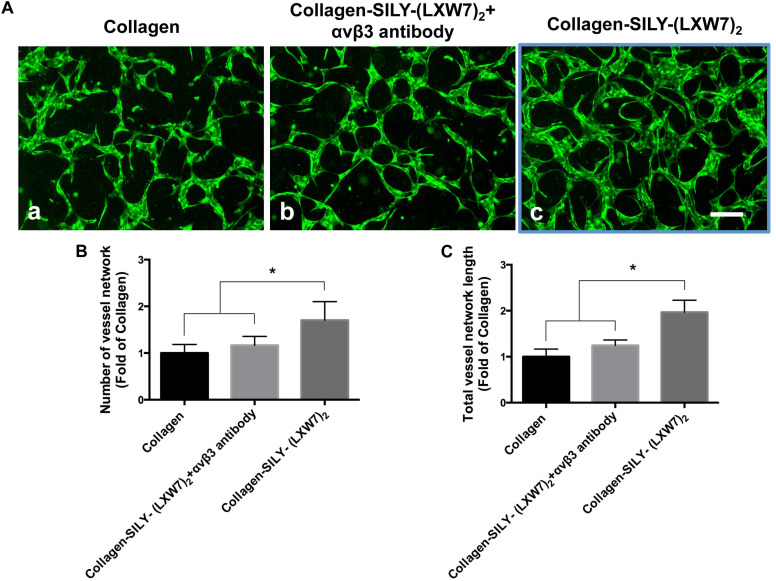
Network formation of ECFCs in LXW7-modified collagen hydrogel was blocked by an anti-αvβ3 integrin blocking antibody. **(A)** Network formation of ECFCs cultured in untreated collagen hydrogel **(a)**, network formation of ECFCs pretreated with an anti-αvβ3 integrin blocking antibody cultured in LXW7-modified collagen hydrogel **(b)** and network formation of ECFCs cultured in LXW7-modified collagen hydrogel **(c)**. Scale bar = 200 μm. **(B)** Quantification of the numbers of vessel network. **(C)** Quantification of the total vessel network length. Data were expressed as mean ± standard deviation: ^∗^*p* < 0.05 (*n* = 5).

Endothelial cell sprouting is important for the EC migration and vascular network formation, which are essential for angiogenesis and vascularization (Blanco and Gerhardt, 2013). Improving EC binding in the hydrogel, by increasing integrin-based binding sites, is vital to promote EC sprouting and branching (Li et al., 2017). The results of ECFC sprouting showed that most of the ECFCs cultured in LXW7-modified collagen hydrogel exhibited long spindle morphology after 1 day culture (Figure 5A,b), but the ECFCs cultured in untreated collagen hydrogel still displayed a round morphology (Figure 5A,a). After 3-day culture, the ECFCs cultured in LXW7-modified collagen hydrogel exhibited obvious sprouting (Figure 5A,d), but only few ECFCs cultured in untreated collagen hydrogel exhibited sprouting (Figure 5A,c). Quantification of cell area (Figure 5B) and number of sprouts (Figure 5C) showed that LXW7 modification significantly improved ECFC spreading and sprouting in collagen hydrogel, indicating that the LXW7-modified collagen hydrogel could promote EC migration and new vascular network formation in vascular tissue regeneration. Capillary morphogenesis is a reliable *in vitro* analog of *in vivo* angiogenesis. Also, it is know that cell-matrix interaction could generate cellular force that can impact capillary network formation. The results of network formation showed the ECFCs cultured in LXW7-modified collagen hydrogel for 5 days formed uniform and integrated network (Figure 6A,c), but the ECFCs cultured in untreated collagen hydrogel (Figure 6A,a) and the anti-αvβ3 integrin antibody blocked ECFCs cultured in LXW7-modified collagen hydrogel (Figure 6A,b) for 5 days only formed few intermittent network and some branches. Quantification of the number of vessel network (Figure 6B) and total vessel network length (Figure 6C) showed the LXW7-modified collagen hydrogel significantly improved the network formation of ECFCs compared to the untreated collagen hydrogel, and the network formation of the ECFCs cultured in LXW7-modified collagen hydrogel was significantly decreased by blocking the anti-αvβ3 integrins using a monoclonal anti-αvβ3 integrin antibody, which indicated that LXW7-modified collagen hydrogel promotes the vascular network formation of ECFCs compared to the untreated collagen hydrogel by increasing integrin αvβ3 binding sites.

### LXW7-Modified Collagen Hydrogel Improved ECFC Proliferation and Survival

**FIGURE 7:**
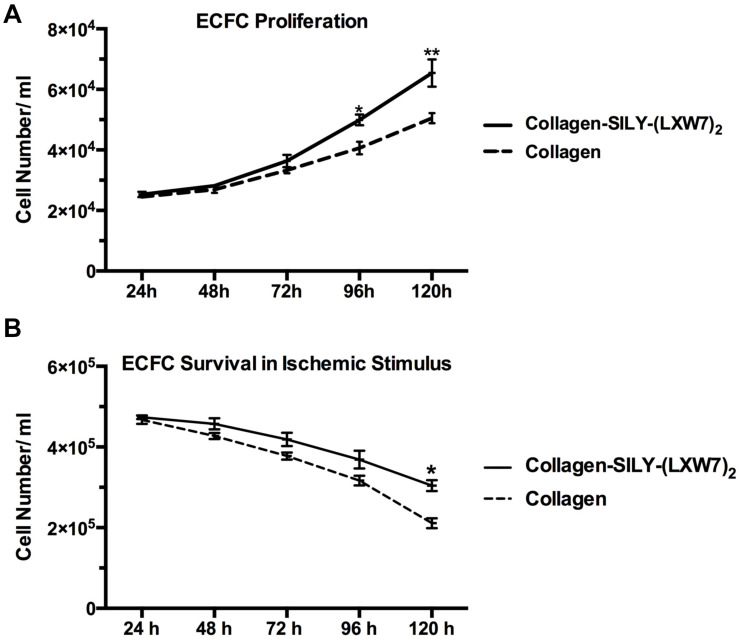
ECFC proliferation and survival in LXW7-modified collagen hydrogel. **(A)** Quantification of cell metabolic activity and proliferation of ECFCs cultured in collagen hydrogel with or without LXW7 modification for 5 days. **(B)** Quantification of survival of ECFCs cultured in collagen hydrogel with or without LXW7 modification under ischemic-mimicking hypoxic environment for 5 days. Data were expressed as mean ± standard deviation: ^∗^*p* < 0.05, ^∗∗^*p* < 0.01 (*n* = 5).

Cell proliferation and survival play key roles in stem cell therapy and tissue regeneration (Lambrou and Remboutsika, 2014; Soteriou and Fuchs, 2018). Integrin-mediated cell binding to the ECM strictly regulates cell cycle progression in mammalian cells, which is crucial for cell proliferation and survival (Schwartz and Assoian, 2001; Vachon, 2011). For ECFC metabolic activity and proliferation evaluation, MTS results showed that the number of ECFCs cultured in LXW7-modified collagen hydrogel was higher, compared to the number of ECFCs cultured in untreated collagen hydrogel, and showed the significant difference after day 4 (Figure 7A). For ECFC survival evaluation, results showed that the number of ECFCs cultured in LXW7-modified collagen hydrogel under the ischemic-mimicking hypoxic environment was significantly higher, compared to the number of ECFCs cultured in untreated collagen hydrogel at day 5 (Figure 7B). Thus, LXW7-modified collagen hydrogel can promote ECFC proliferation and survival and therefore will be beneficial to vascular tissue regeneration.

### LXW7-Modified Collagen Hydrogel Improved ECFC Survival and Engraftment in a Mouse Subcutaneous Implantation Model

**FIGURE 8:**
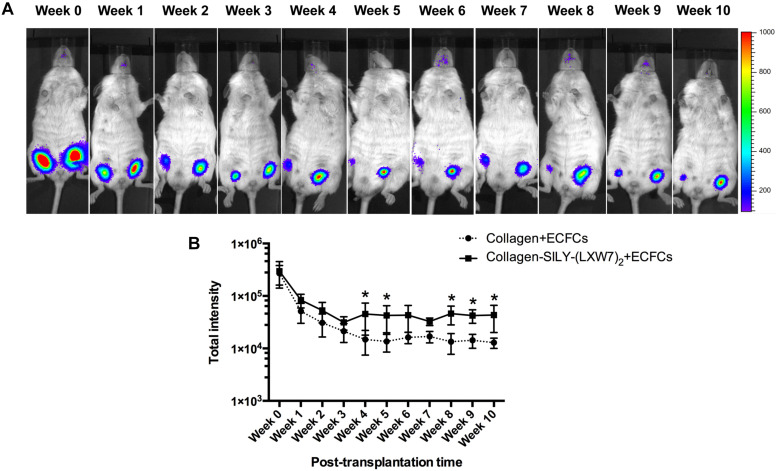
ECFC engraftment in LXW7-modified collagen hydrogel after implantation. **(A)** IVIS imaging of implanted ECFC engraftment in untreated collagen hydrogel (right side on animal) and LXW7-modified collagen hydrogel (left side on animal) at different time points. The belly of animal was facing you in the image. **(B)** Quantification of the luciferase intensity of the engrafted ECFCs in collagen hydrogel with or without LXW7 modification at different time points. Data were expressed as mean ± standard deviation: ^∗^*p* < 0.05 (*n* = 4).

**FIGURE 9:**
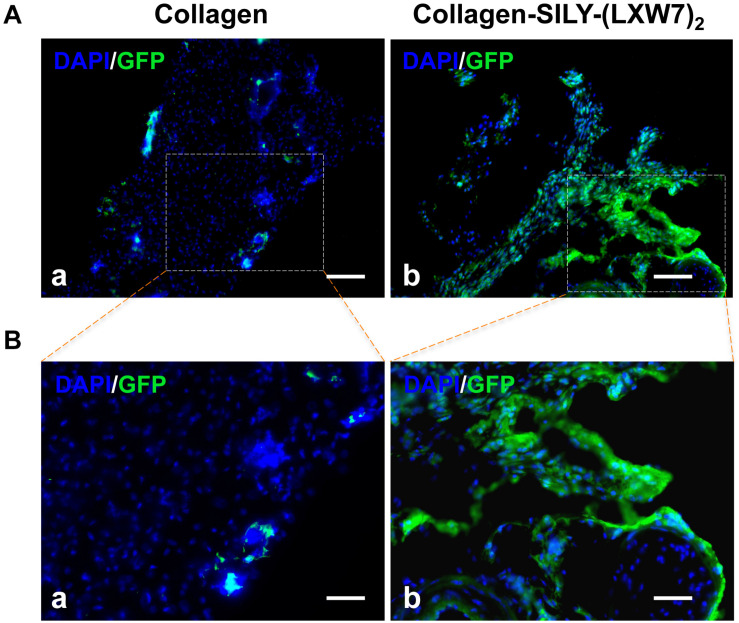
Immunohistological analysis of ECFC survival and engraftment after *in vivo* transplantation. **(A)** GFP staining of implanted ECFCs in untreated collagen hydrogel **(a)** and LXW7-modified collagen hydrogel **(b)** at week 10. **(B)** High magnification imaging of the boxed area of **(A)**.

Different types of hydrogel have been widely studied for cell transplantation (Drury and Mooney, 2003), however, cell survival and engraftment after transplantation remains as one of the key limiting factors for translational applications (Cao et al., 2001). Integrin-ECM interaction is important to regulate cell survival and engraftment (Kumaran et al., 2005). IVIS results showed that LXW7-modified collagen hydrogel prevented the decline in the number of implanted ECFCs at week 1 and improved the engraftment of implanted ECFCs in the next few weeks, compared to the untreated collagen hydrogel (Figure 8A), and further showed the significant difference from week 4, according to the luciferase intensity quantification results (Figure 8B). Because the implanted ECFCs have also been transduced with GFP before implantation, the engraftment of implanted ECFCs was further evaluated using immunohistological staining with anti-GFP antibody. The immunohistological staining results showed that LXW7-modified collagen hydrogel promoted the ECFC survival and engraftment, compared to the untreated collagen hydrogel, and some ECFCs formed vascular network structures in the LXW7-modified collagen hydrogel construct (Figure 9). Thus, LXW7-modified collagen hydrogel represents a good candidate for promoting ECFC engraftment and holds promise for improving vascularization and tissue regeneration. It is known that neovascularization plays the key role in many different kinds of tissue regeneration, therefore, the LXW7-functionalized collagen hydrogel developed in this study could be widely used as a novel injectable EC delivery biomaterial tool for a variety of tissue regeneration applications, including hindlimb ischemia, myocardial ischemia and so on. Therefore, in this proof-of-concept study, we chose to use a more generic but widely applicable subcutaneous implantation model to evaluate the function of the novel LXW7-modified collagen hydrogel on neovascularization. Data obtained from this model have broad implications for a wide range of tissue regeneration applications. Further detailed evaluation of the functions of LXW7-modified collagen hydrogel in specific disease models is warranted in future studies.

## Conclusion

This study sought to engineer collagen hydrogel, a widely used ECM biomaterial hydrogel, to improve its function for EC transplantation and tissue regeneration. To improve the EC-matrix interaction, we engineered the collagen hydrogel by increasing EC specific integrin binding sites. We successfully developed a technology to molecularly conjugate an integrin αvβ3 ligand LXW7 onto the collagen backbone via “SILY-collagen” binding approach. The LXW7-modified collagen hydrogel exhibited the capacities for promoting EC survival in an ischemic-mimicking environment *in vitro* and improved the engraftment of transplanted ECs and supported ECs to form functional vascular network structures *in vivo*. The LXW7-functionalized collagen hydrogel holds the promise to be used as a novel EC delivery tool and injectable biomaterial for tissue engineering and regenerative medicine.

## Data Availability Statement

All datasets generated for this study are included in the article/Supplementary Material.

## Ethics Statement

The animal study was reviewed and approved by Institutional Animal Care and Use Committee at the University of California, Davis.

## Author Contributions

DH performed the construction and the *in vitro* and *in vivo* evaluation of LXW7-modified collagen hydrogel, wrote the manuscript, and discussed the results. RL performed the synthesis of (SILY)_2_-LXW7 and SILY-(LXW7)_2_, performed the HPLC analysis, and discussed the results. KG assisted the *in vivo* evaluation. CH, SH, and CZ assisted the *in vitro* evaluation. GS, DF, AP, and KL were involved in the results discussion. AW was responsible for conceptualization, results, discussion, and revision of the manuscript. All authors contributed to the article and approved the submitted version.

## Conflict of Interest

The authors declare that the research was conducted in the absence of any commercial or financial relationships that could be construed as a potential conflict of interest.
