# Supplementary material for: Developing an Injectable Nanofibrous Extracellular Matrix Hydrogel With an Integrin αvβ3 Ligand to Improve Endothelial Cell Survival, Engraftment and Vascularization
Source: Front Bioeng Biotechnol. 2020 Jul 29;8:890. doi: 10.3389/fbioe.2020.00890 (PMC7403189; doi:10.3389/fbioe.2020.00890)
Supplement: Supplementary file 1 [file Data_Sheet_1.PDF]

Supplementary Material

Synthesis of SILY-(LXW7)$_2$

Synthesis of SILY-(LXW7)$_2$ involved in three steps: solid phase synthesis of SILY-2N_3, solid phase synthesis of LXW7-DBCO; DBCO-azido copper-free click conjugation.

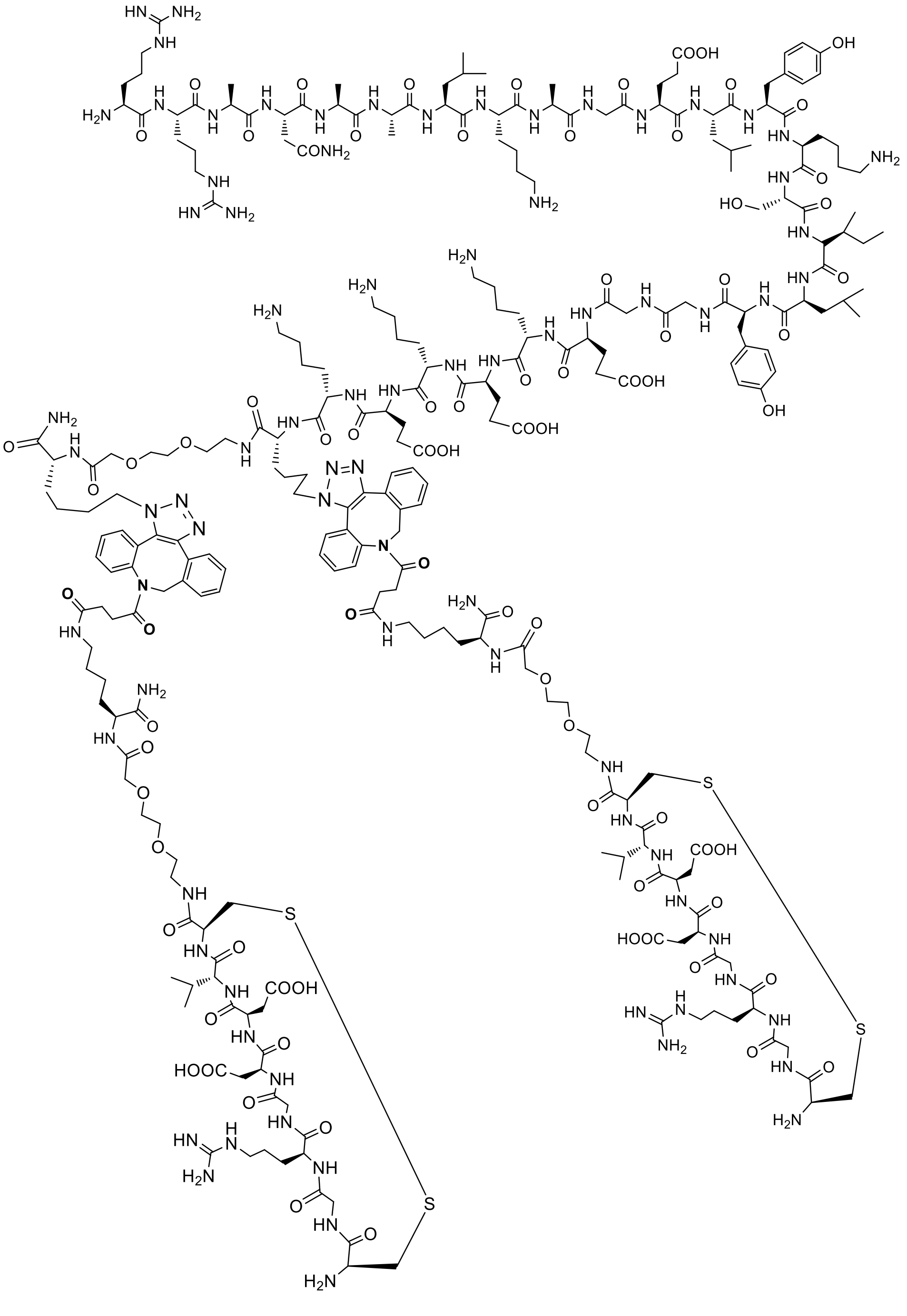

Chemical structure of SILY-(LXW7)$_2$

Step 1. Solid phase synthesis of SILY-2N_3

SILY-2N_3 contains the peptide sequence RRANAALKAGELYKSILY-GGEKEKEK-K(N_3)-AEEA linker-K(N_3) whereas GGEKEKEK is a stealth linker and AEEA is a short PEG linker. The synthetic approach is shown in Scheme x. The peptide was synthesized on Rink amide MBHA resin (loading 0.503 mmol/g) and cleaved off the beads with Reagent K cleavage cocktail of 82.5% trifluoroacetic acid (TFA): 5% thioanisole: 5% phenol:5% water: 2.5% triisopropylsilane (TIS) (v/v). Fmoc-amino acid coupling (5 eq. to beads) was achieved using HCTU (5 eq.) and DIEA (10 eq.) as coupling reagents. The coupling was carried out at room temperature for 2-5 h. Fmoc was deprotected with 20% 4-methylpiperidine in DMF twice (5 and 15 minutes, respectively). The crude peptide was precipitated with cold ether and purified with Reverse Phase High Performance Liquid Chromatography (RP-HPLC). The structure is verified by ESI-MS: 1688.45 (m/z=2), 1125.97 (m/z=3), 844.73 (m/z=4), 675.98 (m/z=5), 563.48 (m/z=6).

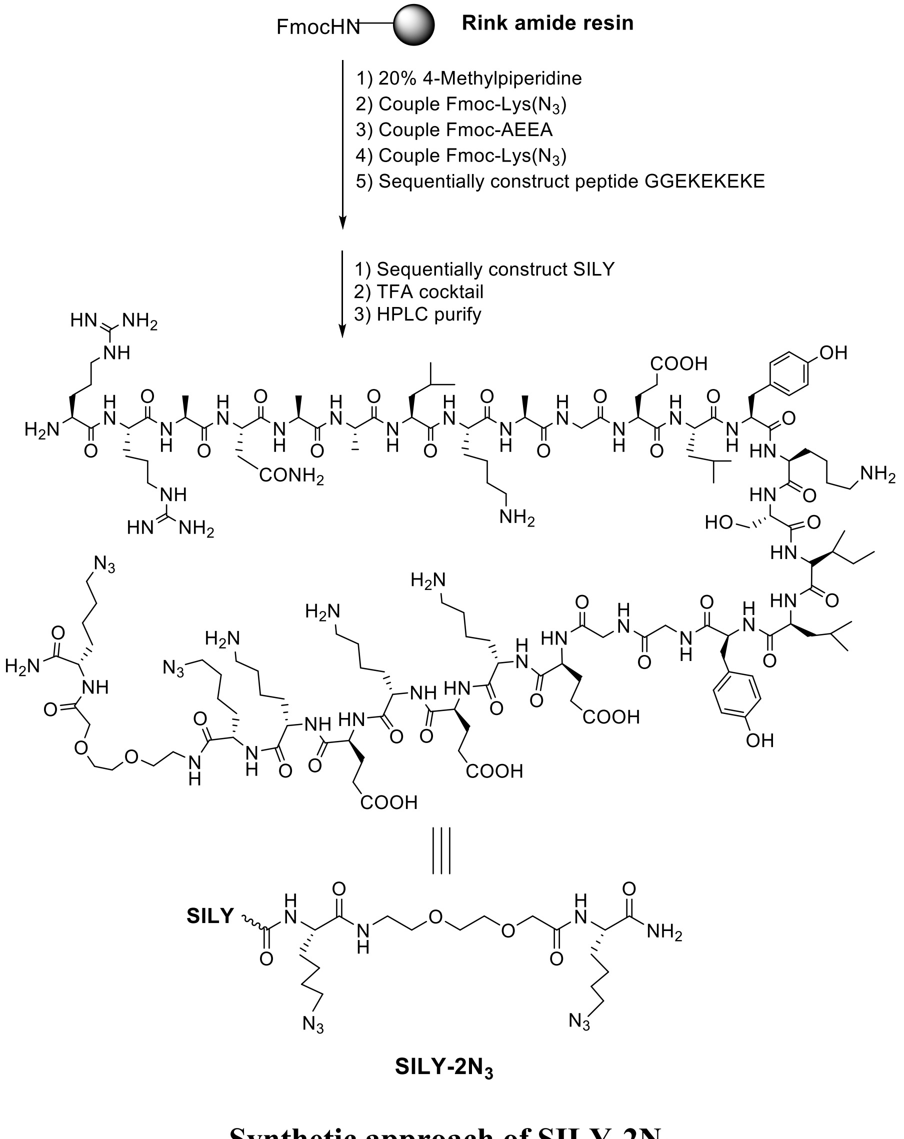

SILY-2N_3

Synthetic approach of SILY-2N_3

Step 2. Solid phase synthesis of LXW7-DBCO

Fmoc-LXW-AEEA-k was first synthesized using standard SPPS approach and cleaved off the beads using TFA cocktail. The crude linear peptide was cyclized in solution with CLERA-OX resin. DBCO was coupled the side chain of D-lysine (k) at the C-terminal using DBCO-NHS. After Fmoc was removed with 25% 4-methylpiperidine, the crude product was submitted to HPLC purification and lyophilization, LXW7-DBCO was obtained as an off-white powder. MALDI-TOF MS: Found: 1381.85. Calculated: 1381.57 $[M+H]^+$.

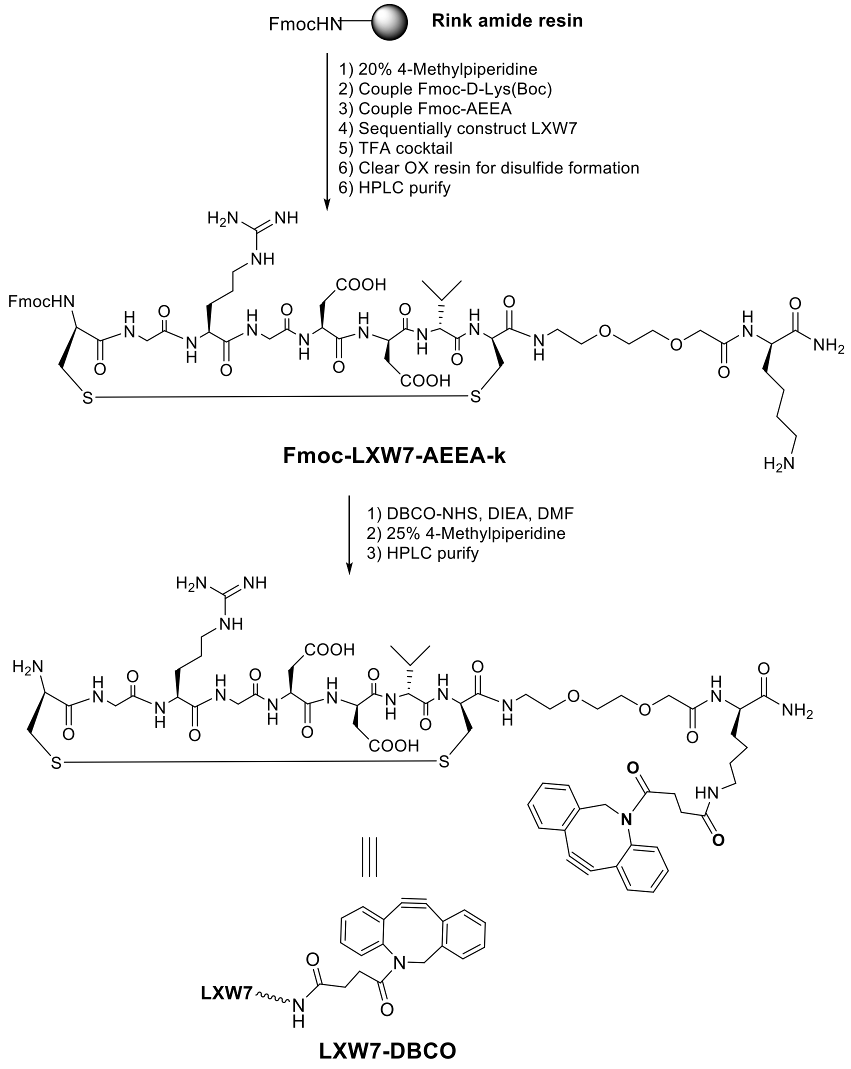

Synthesis of LXW7-DBCO

Step 3. Copper-free Click conjugation

Dissolved SILY-2(N_3) and LXW7-DBCO in a mole ratio of 1:2 in a mixture of 50% acetonitrile (ACN) in water and stirred at room temperature overnight. The resulting solution was submitted for purification with RP-HPLC; Vydac C18 column (10 µm, 22 x 250 mm). The structure was verified by MALDI-TOF MS.

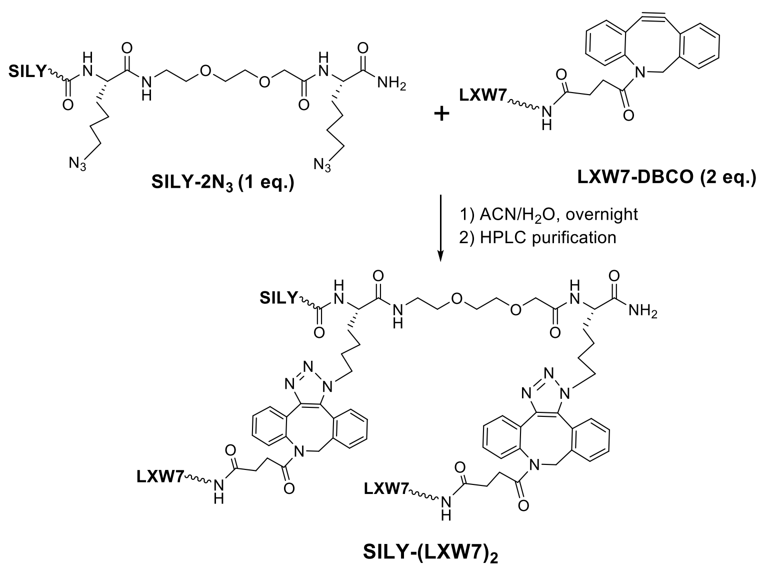

Copper-free Click conjugation of SILY-(LXW7)$_2$

Synthesis of LXW7-(SILY)$_2$

Synthesis of LXW7-(SILY)2 also involved in three steps: solid phase synthesis of LXW7-2N_3, solid phase synthesis of SILY-DBCO; DBCO-azido copper-free click conjugation.

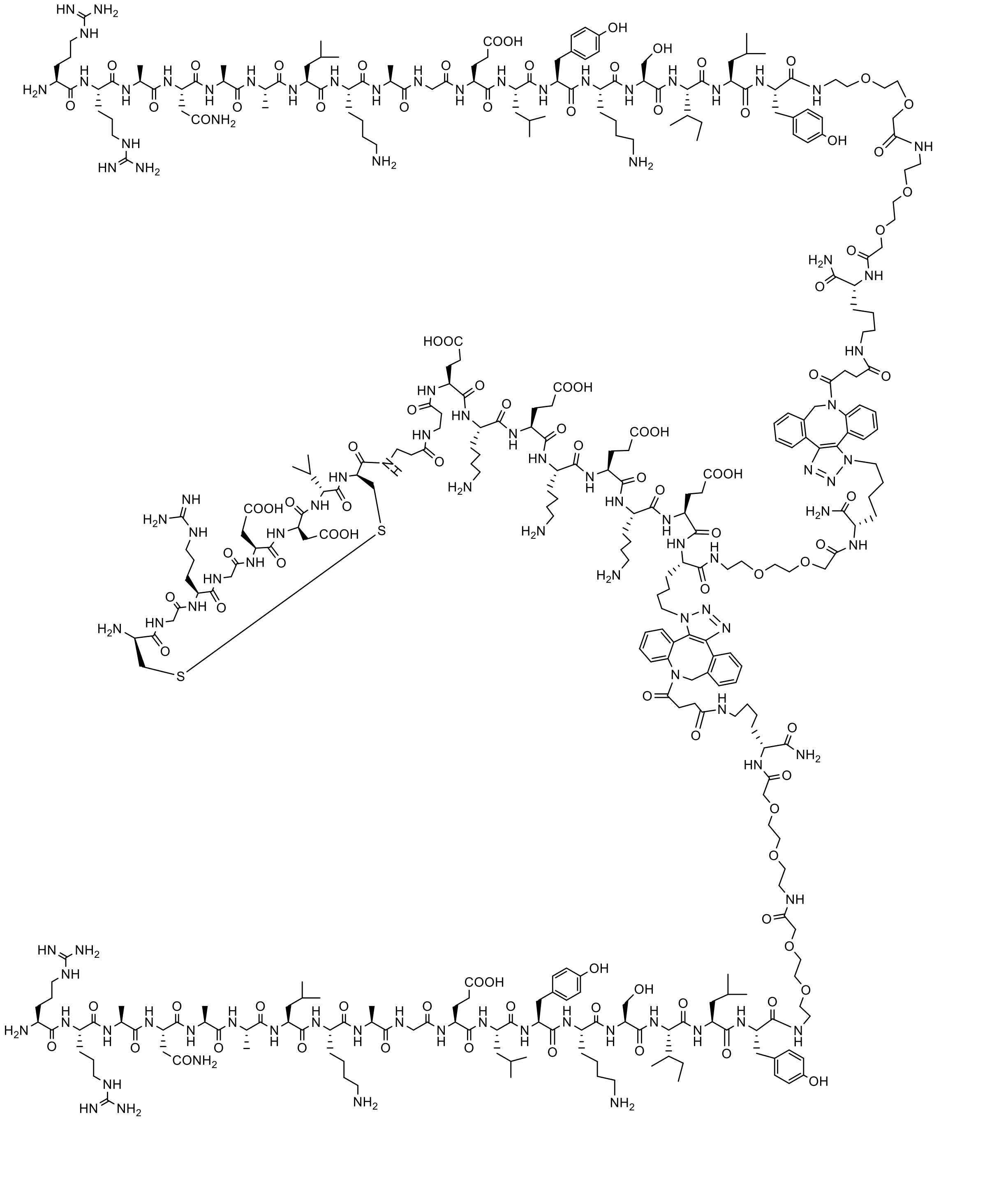

Chemical structure of LXW7-(SILY)$_2$

Step 1. Solid phase synthesis of LXW7-2N_3

LXW7-2N_3 contains the peptide sequence of cGRGDdvc-BA-BA-EKEKEKE-K(N_3)-AEEA-K(N_3) whereas BA is beta alanine and AEEA is a short PEG linker. EKEKEKE was chosen as a stealth linker. LXW7-2N_3 was synthesized using similar approach as described above.

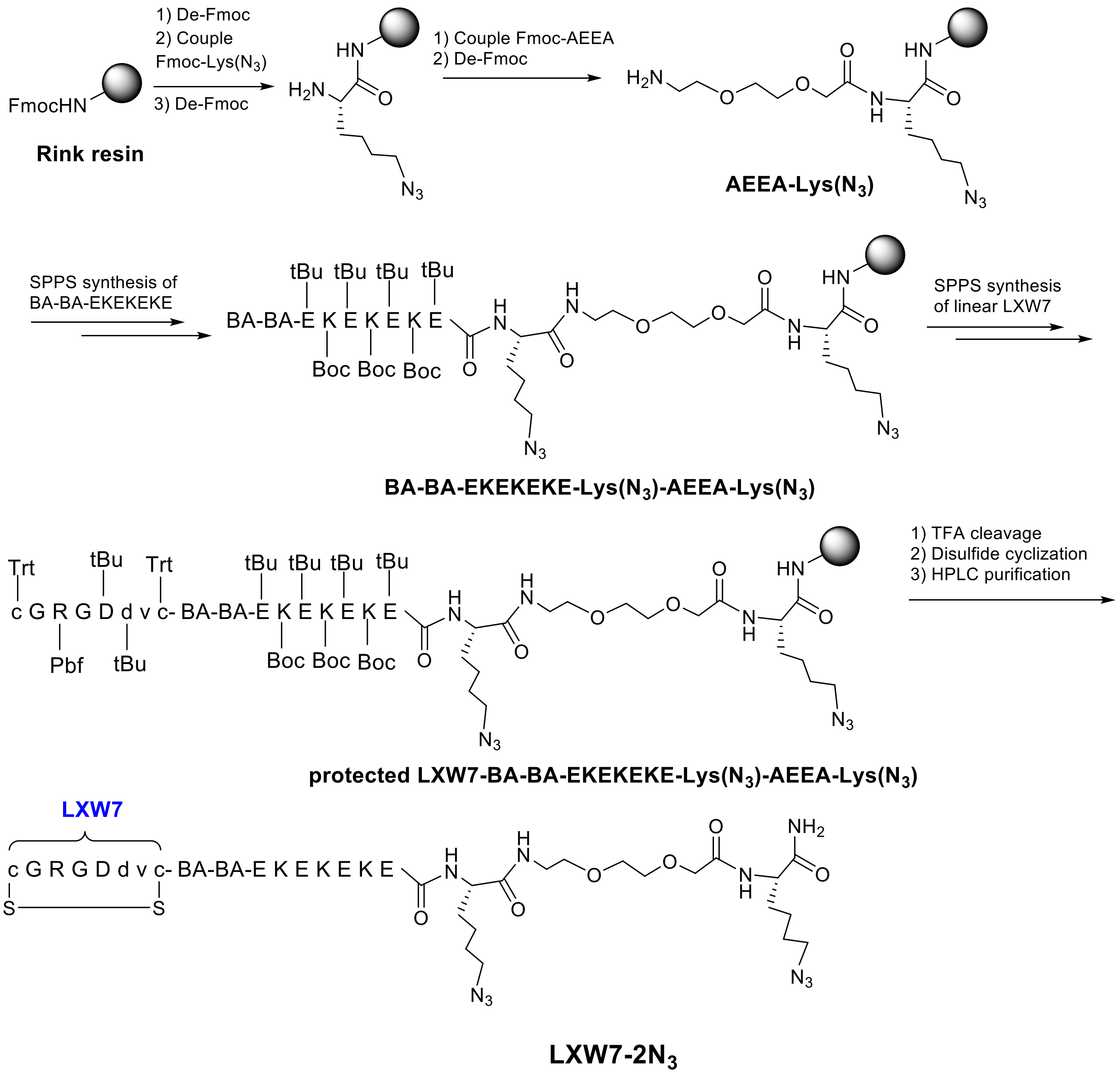

Synthetic approach of LXW7-2N_3

Step 2. Solid phase synthesis of SILY-DBCO

The synthesis was achieved using similar approach as LXW7-DBCO.

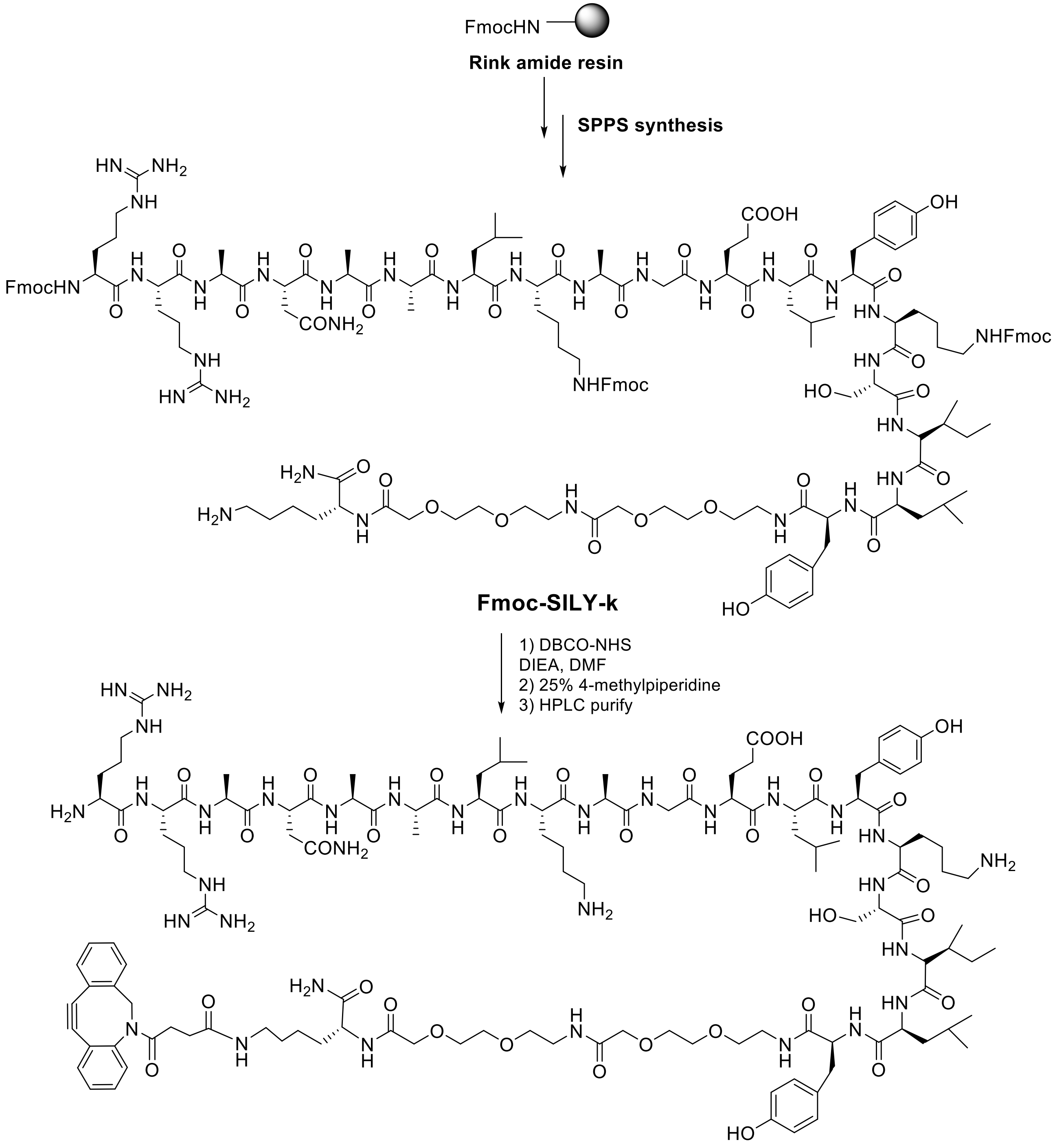

SILY-DBCO

Synthetic approach of SILY-DBCO

Step 3. Copper-free Click conjugation

The synthetic approach is shown in Fig S8. Dissolved LXW7-2(N_3) and SILY-DBCO in a mole ratio of 1:2 in a mixture of 50% acetonitrile (ACN) in water and stirred at room temperature overnight. The resulting solution was submitted for purification with RP-HPLC; Vydac C18 column (10 µm, 22 x 250 mm). The structure was verified by MALDI-TOF MS.

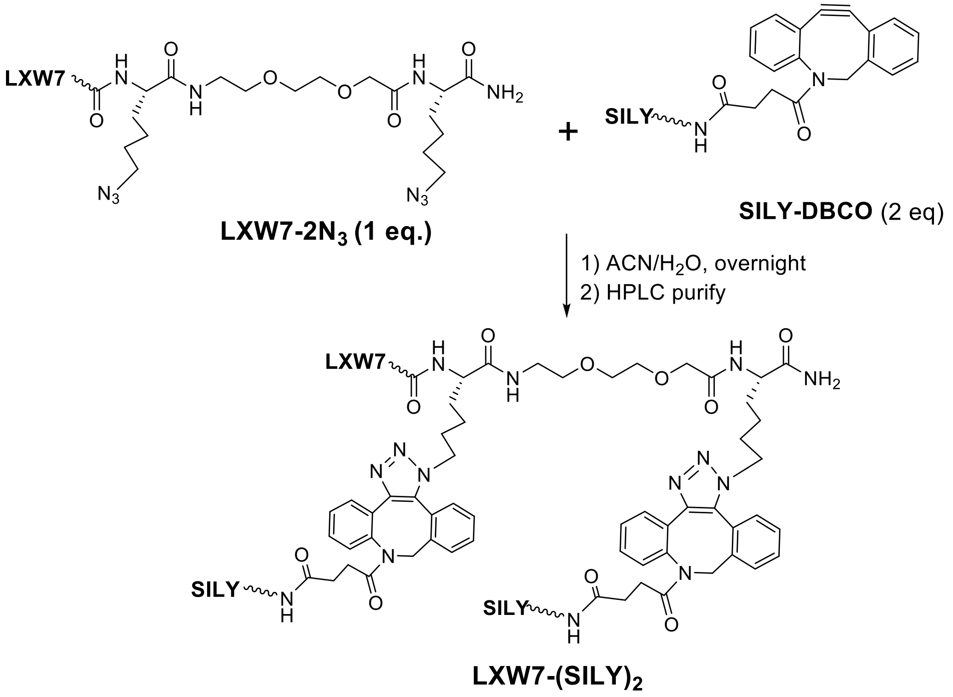

Copper-free Click conjugation of LXW7-(SILY)$_2$

Supplemental Figure 1. Chemical structures of SILY-(LXW7)$_2$ and (SILY)$_2$-LXW7.

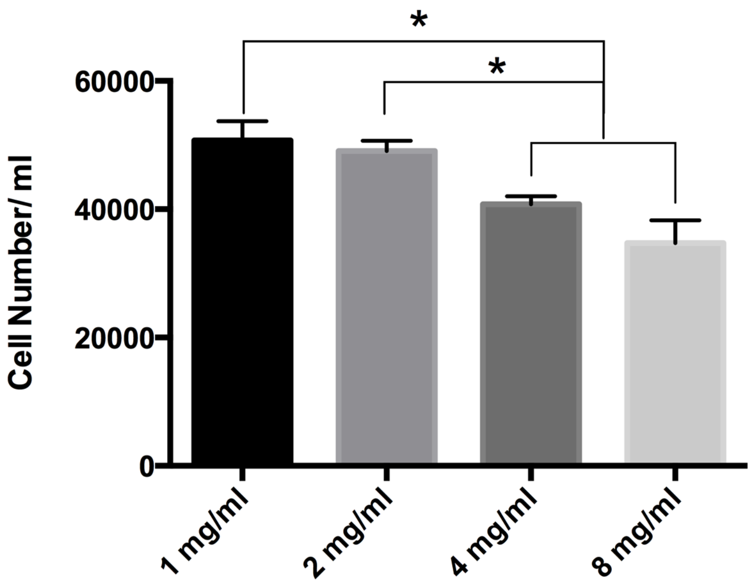

Supplemental Figure 2. Optimal concentration of collagen hydrogel for ECFC growth. Data were expressed as mean ± standard deviation: *$p < 0.05$ ($n = 5$).

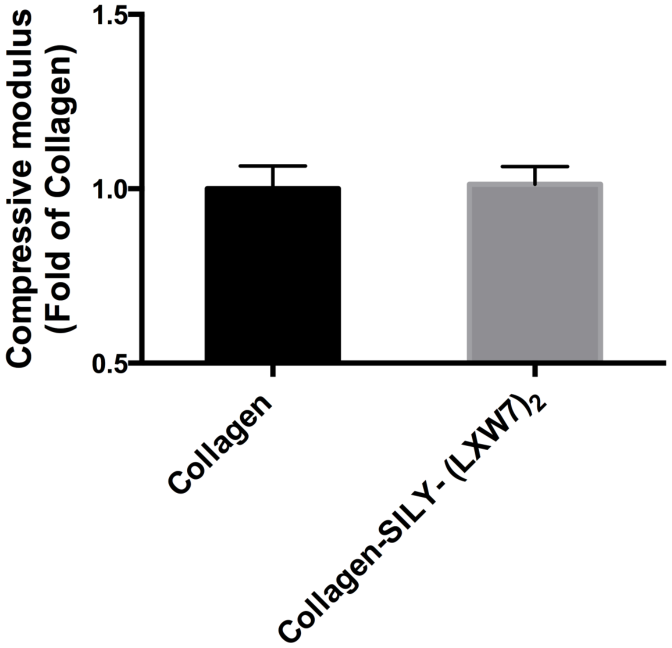

Supplemental Figure 3. Compressive modulus of collagen hydrogel before and after LXW7 modification. Data were expressed as mean ± standard deviation, and no significant difference between two groups.
